# Sleep Disturbance Patterns in Children with Leukemia and Lymphoma: Domain-Specific Associations with Hospitalization and Treatment Context

**DOI:** 10.3390/jcm15155785

**Published:** 2026-07-23

**Authors:** Mehtap Ertekin, Salih Gozmen

**Affiliations:** Department of Pediatric Hematology and Oncology, Izmir City Hospital, Şevket Ince Mahallesi, 2148/11 Sokak, No. 1/11, Bayraklı, 35540 Izmir, Türkiye; drsalihgozmen@gmail.com

**Keywords:** pediatric oncology, leukemia, lymphoma, sleep disturbance, Children’s Sleep Habits Questionnaire, hospitalization, corticosteroids, parental education

## Abstract

**Objectives:** The importance of sleep disturbance as a component of supportive care in pediatric oncology has recently been recognized, but the characteristics of sleep for this particular disease category have yet to be elucidated. **Methods:** This cross-sectional study included 54 pediatric patients diagnosed with leukemia or lymphoma and 56 age- and sex-matched clinically stable children attending routine pediatric hematology outpatient follow-up for benign hematological conditions. Sleep was assessed using the Children’s Sleep Habits Questionnaire (CSHQ). Global and domain-specific sleep scores were compared. Multivariable linear regression was used to identify correlates of global sleep disturbance, including parental educational attainment. Within the patient group, recent hospitalization and corticosteroid exposure were modeled separately because of strong collinearity. **Results:** Patients had higher global CSHQ scores than the comparison group (53.56 ± 8.28 vs. 49.07 ± 8.09, *p* = 0.005; Cohen’s d = 0.55). Differences were most evident in sleep anxiety and parasomnias (exploratory subscale findings), whereas sleep duration did not differ significantly. In the whole-sample model, patient status (B = 4.16, *p* = 0.006) and higher parental educational attainment (B = −2.13, *p* < 0.001) were independently associated with global sleep disturbance. Within the patient group, recent hospitalization was the strongest clinical correlate of sleep burden (B = 11.18, *p* < 0.001; R^2^ = 0.433). Corticosteroid exposure was associated with higher sleep disturbance only in models excluding hospitalization. **Conclusions:** Children with leukemia and lymphoma showed a selective parent-reported sleep disturbance pattern characterized mainly by sleep anxiety and parasomnias rather than reduced sleep duration. Recent hospitalization and parental educational attainment appear to be important correlates of sleep burden in pediatric hematology-oncology care.

## 1. Introduction

Sleep is a fundamental component of child health and is essential for neurodevelopment, emotional regulation, cognitive performance, and immune functioning. Even modest sleep disruption may adversely affect daytime behavior, school functioning, family well-being, and overall quality of life. Sleep disorders are prevalent among children undergoing cancer treatment and survivors, but they are generally neglected in medical practice since the focus is usually on managing the disease and its side effects [1,2,3,4,5,6,7,8].

Sleep problems can be a risk for children suffering from hematologic malignancy. This is because factors such as frequent hospital admissions, long-term effects of treatment, monitoring at night, drugs, and worries associated with illnesses and treatment might cause sleeplessness. In the current setting, corticosteroids become especially significant as they form an important part of several protocols for leukemia and lymphomas and have the potential to affect sleep via their influence on circadian rhythms, hypothalamic–pituitary–adrenal axis functioning and central nervous system arousal [9,10,11,12,13,14].

Even though there are many studies that have been carried out before on pediatric oncology, most of the literature that exists today focuses on the overall sleep burden among cancer patients rather than establishing sleep profiles specific to certain domains within hematological cancers. As a result, it remains unclear whether sleep disruption in children with leukemia and lymphoma is primarily characterized by shortened sleep duration, sleep fragmentation, anxiety-related disturbances, parasomnias, or a broader generalized pattern [6,7,8,11,14,15].

The present study therefore aimed to compare global and domain-specific sleep disturbances in children with leukemia or lymphoma and a clinically stable benign hematology outpatient comparison group, and to examine whether demographic characteristics, recent hospitalization, and corticosteroid exposure were associated with global sleep disturbance. We hypothesized that patients would demonstrate higher global sleep disturbance than the comparison group, with selective elevation in anxiety-related and parasomnia-related domains, and that recent hospitalization and corticosteroid exposure would be associated with greater sleep disturbance within the patient group.

## 2. Materials and Methods

### 2.1. Study Design and Participants

This cross-sectional controlled observational study was conducted in May 2026, following ethics committee approval. This study included 54 children diagnosed with leukemia or lymphoma and 56 children in a benign hematology outpatient comparison group. Patients were recruited from a tertiary pediatric hematology-oncology center during outpatient follow-up or treatment-related clinical assessment. Eligibility criteria for the patient group included a confirmed diagnosis of leukemia or lymphoma, age between 2 and 16 years, ongoing treatment or follow-up within the pediatric hematology-oncology program, and availability of complete questionnaire data. During the data collection period, 67 children with leukemia or lymphoma were initially screened for eligibility, of whom 13 were excluded because of incomplete questionnaire data or failure to meet the eligibility criteria above, leaving 54 patients in the final analytic sample. Similarly, 61 children in the benign hematology outpatient comparison group were initially screened, of whom 5 were excluded because of incomplete questionnaire data, leaving 56 comparison-group children in the final analytic sample. Children with known primary sleep disorders predating their malignancy diagnosis, severe neurodevelopmental disorders, or acute critical illness at the time of assessment were excluded.

The comparison group consisted of clinically stable children with benign hematological conditions, such as iron deficiency anemia or thalassemia minor, who were followed in the pediatric hematology outpatient clinic. These children had no prior diagnoses of oncological disorders, infections, fever, systemic disorders, or primary sleep disorders, and had no regular use of medications or hospital admission in the preceding eight weeks. However, because this group was selected from children followed for benign hematological conditions rather than from an entirely healthy population, it should be regarded as a baseline with its own mild, condition-related sleep vulnerabilities rather than as a group entirely free of sleep-affecting factors. Comparison subjects were matched for age and sex with the patient population.

### 2.2. Sleep Assessment

Sleep disturbance was measured by the Children’s Sleep Habits Questionnaire (CSHQ) [16,17], which is an already established parental measure with 33 items on a 3-point Likert scale. The Turkish-validated version of the CSHQ was used in this study. According to the recommended scoring process, items Q1, Q2, Q3, Q10, Q11, and Q26 were reverse-scored such that high scores represented more sleep problems. A global CSHQ score was calculated by adding up all items, including subscales of bedtime resistance, sleep onset latency, sleep anxiety, total sleep time, night awakenings, parasomnias, breathing-related sleep disorders, and sleepiness during the day. Because the CSHQ is completed by parents, scores were interpreted as parent-reported sleep disturbance burden rather than objectively confirmed sleep disruption.

### 2.3. Clinical Variables

Within the patient group, recent hospitalization during the preceding eight weeks and systemic corticosteroid exposure during the preceding eight weeks were recorded. Corticosteroid exposure was defined as protocol-based systemic corticosteroid treatment during this period. In patients with leukemia and non-Hodgkin lymphoma who received corticosteroids, dexamethasone was used at a dose of 20 mg/m^2^/day according to the relevant treatment protocol. In patients with Hodgkin lymphoma, prednisolone was used at a dose of 60 mg/m^2^/day. Because of the limited number of patients in each diagnostic and corticosteroid subgroup, corticosteroid exposure was analyzed primarily as present or absent, while corticosteroid agent and protocol-based dose were reported descriptively.

### 2.4. Statistical Analysis

Analyses were performed using SPSS v22. Normally distributed continuous variables were assessed by distribution graphs and Shapiro–Wilk tests. Group comparisons between patients and the comparison group were performed with independent samples *t*-tests for continuous variables and chi-square tests for categorical variables. Effect sizes were reported as Cohen’s d.

To identify independent predictors of global sleep disturbance, multivariable linear regression models were constructed with total CSHQ score as the dependent variable. In the whole-sample model, patient status was entered as the primary predictor, while adjusting for age and sex as covariates. Within the patient group, hospitalization status and corticosteroid exposure were analyzed in separate models because of strong collinearity (entering both variables simultaneously would have produced severe multicollinearity and unacceptably inflated variance inflation factors, given their near-complete overlap described in the Section 3); a combined model was examined only as a secondary descriptive analysis and was not considered inferential. Regression results are reported as unstandardized B coefficients with 95% confidence intervals. Two-sided *p* values < 0.05 were considered statistically significant. The underlying assumptions of linear regression, including normality of residuals and homoscedasticity, were checked and were adequately met for all models reported. Because subscale analyses were exploratory, *p* values for those comparisons were not adjusted for multiple testing and were interpreted alongside effect sizes.

No formal a priori sample size calculation was performed. Therefore, the study should be interpreted as exploratory, particularly with regard to subgroup comparisons and multivariable regression analyses. Regression models were planned a priori to include a limited, clinically relevant set of covariates in order to maintain an adequate observations-per-predictor ratio; the patient-only models (three predictors, *n* = 54) adequately meet and exceed the conventional 10-to-15 observations-per-predictor benchmark, minimizing the risk of overparameterization. For treatment-phase comparisons, post hoc pairwise comparisons were performed with Bonferroni correction.

As a secondary descriptive analysis, the proportion of children exceeding a previously proposed CSHQ screening threshold of total CSHQ score ≥ 41 was compared between patients and the comparison group using chi-square testing, with odds ratios and 95% confidence intervals calculated where estimable. Fisher’s exact test was applied when expected cell counts were small.

Parental educational attainment was recorded as a four-level ordinal variable (primary school, middle school, high school, university) and was examined as a potential sociodemographic covariate. One-way ANOVA was used to assess differences in global sleep disturbance across educational groups, with Bonferroni-corrected pairwise comparisons for post hoc testing. Parental education was subsequently entered as an additional predictor in the whole-sample regression model to evaluate its independent contribution.

## 3. Results

A total of 110 children were included in the analysis, comprising 54 patients with leukemia or lymphoma and 56 clinically stable children in the benign hematology outpatient comparison group. The two groups were comparable in terms of age (7.58 ± 3.87 vs. 8.44 ± 3.52 years, *p* = 0.229) and sex distribution (male: 50.0% vs. 48.2%; female: 50.0% vs. 51.8%, *p* = 1.000) (Table 1). Within the patient group, 21 children (38.9%) had received corticosteroids and 29 (53.7%) had been hospitalized within the preceding eight weeks. Corticosteroid exposure and recent hospitalization were strongly associated (χ^2^(1) = 29.6, *p* < 0.001; φ = 0.741) and both were concentrated almost exclusively in the intensive therapy phase, confirming that they should be interpreted primarily as markers of treatment intensity (see Table 1 footnote for full cross-tabulations).

Parental education did not differ significantly between groups (χ^2^(3) = 1.52, *p* = 0.677). Recent hospitalization was concentrated in the intensive therapy phase: all children receiving intensive therapy had been recently hospitalized (28/28, 100%), compared with none in the maintenance phase (0/10, 0%) and one in the off-treatment group (1/16, 6.2%; χ^2^(2) = 50.23, *p* < 0.001). Corticosteroid exposure followed a similar pattern, occurring predominantly during intensive therapy (21/28, 75.0%) and not at all during maintenance (0/10, 0%) or off-treatment follow-up (0/16, 0%; χ^2^(2) = 31.91, *p* < 0.001). These distributions indicate that both recent hospitalization and corticosteroid exposure largely served as markers of the intensive therapy phase. Notably, because 100% of steroid-exposed patients had also been recently hospitalized, a combined multi-level model incorporating both exposures simultaneously was not mathematically estimable, owing to empty cells in the crossed exposure categories. This complete overlap reflects institutional treatment protocols, in which corticosteroids are administered concomitantly with other chemotherapeutic agents during inpatient admission and are not given in the outpatient setting; consequently, corticosteroid exposure and recent hospitalization could not occur independently of one another in this cohort.

Within the patient group, 32 children (59.3%) had leukemia and 22 (40.7%) had lymphoma (16 non-Hodgkin, 6 Hodgkin; see Table 1). At the time of assessment, 28 patients (51.9%) were receiving intensive therapy, 10 (18.5%) were in the maintenance phase, and 16 (29.6%) had completed treatment and were in the off-treatment follow-up period.

Global sleep disturbance was significantly greater in patients than in the comparison group. Mean total CSHQ score was 53.56 ± 8.28 in the patient group and 49.07 ± 8.09 in the comparison group, corresponding to a mean difference of 4.49 points (*p* = 0.005; Cohen’s d = 0.55) (Table 2).

Subscale comparisons showed a selective rather than uniform pattern of between-group differences. Patients had significantly higher scores for sleep anxiety (7.81 ± 2.34 vs. 6.82 ± 2.11, *p* = 0.018; Cohen’s d = 0.45) and parasomnias (11.98 ± 3.08 vs. 10.41 ± 2.76, *p* = 0.006; Cohen’s d = 0.54). In contrast, no significant differences were observed for bedtime resistance, sleep onset delay, sleep duration, night wakings, sleep-disordered breathing, or daytime sleepiness (all *p* > 0.05) (Table 2). As these subscale comparisons were not adjusted for multiple testing, they should be interpreted as exploratory patterns requiring confirmation rather than definitive between-group differences.

As a secondary exploratory analysis, global sleep disturbance was compared across treatment phases within the patient group. One-way ANOVA revealed a significant between-phase difference in total CSHQ score (F(2,51) = 10.92, *p* < 0.001). Children receiving intensive therapy had substantially higher sleep disturbance scores than those in the off-treatment group (61.61 ± 6.03 vs. 46.75 ± 7.67; Cohen’s d = 1.42, *p* < 0.001), whereas the off-treatment group did not differ significantly from the comparison group (46.75 ± 7.67 vs. 49.07 ± 8.09; *p* = 0.368, d = 0.30). Children in the maintenance phase showed intermediate scores (57.40 ± 4.99) that did not differ significantly from either the intensive or off-treatment groups after pairwise comparison (*p* = 0.070 and *p* = 0.064, respectively).

In the multivariable linear regression model including the whole sample, patient status, age, sex, and parental educational attainment were entered as predictors. The model was statistically significant (R^2^ = 0.225, adjusted R^2^ = 0.195; F(4,104) = 7.53, *p* < 0.001). Patient status independently predicted greater sleep disturbance (B = 4.158, 95% CI: 1.242 to 7.075, *p* = 0.006), and male sex was inversely associated with total CSHQ score (B = −3.166, 95% CI: −6.084 to −0.247, *p* = 0.034). Age was not a significant predictor (*p* = 0.239). Parental educational attainment was independently and inversely associated with global sleep disturbance (B = −2.131, 95% CI: −3.287 to −0.976, *p* < 0.001), indicating that higher parental education was associated with lower CSHQ scores regardless of patient status (Table 3, Panel A).

Parental educational attainment also differed significantly in its association with global sleep disturbance when examined categorically. One-way ANOVA revealed a significant between-group difference in total CSHQ score by parental education level across the whole sample (F(3,105) = 6.20, *p* < 0.001). Post hoc comparisons with Bonferroni correction indicated that children whose parents had university-level education had significantly lower global sleep disturbance scores than those with primary school education (mean difference = −6.7 points; *p* = 0.003; Cohen’s d = 0.85) and middle school education (mean difference = −10.7 points; *p* = 0.004; Cohen’s d = 1.73). No significant pairwise differences were observed among the remaining groups after correction for multiple comparisons.

In a separate patient-group model excluding hospitalization, corticosteroid exposure was also associated with higher global sleep disturbance (B = 9.543, *p* < 0.001). Male sex remained inversely associated with CSHQ score (B = −5.656, *p* = 0.017), whereas age was again not significant (*p* = 0.272). This model explained 26.7% of the variance (R^2^ = 0.267) (Table 3, Panel C).

Using a previously proposed CSHQ screening threshold of total score ≥ 41, a higher proportion of children with leukemia or lymphoma exceeded this cutoff compared with the benign hematology outpatient comparison group (50/54, 92.6% vs. 43/56, 76.8%; χ^2^(1) = 5.26, *p* = 0.022; OR = 3.78, 95% CI: 1.15–12.45). Because the CSHQ is a parent-reported screening instrument rather than a diagnostic test, and because a substantial proportion of comparison children also exceeded this threshold, this analysis was interpreted as supportive and descriptive. The primary interpretation was therefore based on continuous total and domain-specific CSHQ scores.

Within the patient group, all hospitalized children exceeded the CSHQ screening threshold (29/29, 100%), compared with 84.0% of non-hospitalized patients (21/25; Fisher’s exact *p* = 0.040). Given the complete separation in the hospitalized group, an odds ratio could not be reliably estimated, and this comparison is therefore presented descriptively. By treatment phase, the proportion exceeding the CSHQ screening threshold was highest in children receiving intensive therapy (28/28, 100%), followed by those in the maintenance phase (9/10, 90.0%) and children who had completed treatment (13/16, 81.2%).

Exploratory unadjusted subscale analyses within the patient group showed that corticosteroid-exposed children had higher sleep anxiety scores (9.95 ± 2.06 vs. 8.27 ± 2.53, *p* = 0.014; Cohen’s d = 0.71) and higher night waking scores (5.86 ± 1.28 vs. 4.55 ± 1.48, *p* = 0.002; Cohen’s d = 0.93) than non-exposed patients, whereas daytime sleepiness did not differ significantly (*p* = 0.357).

**Figure 1:**
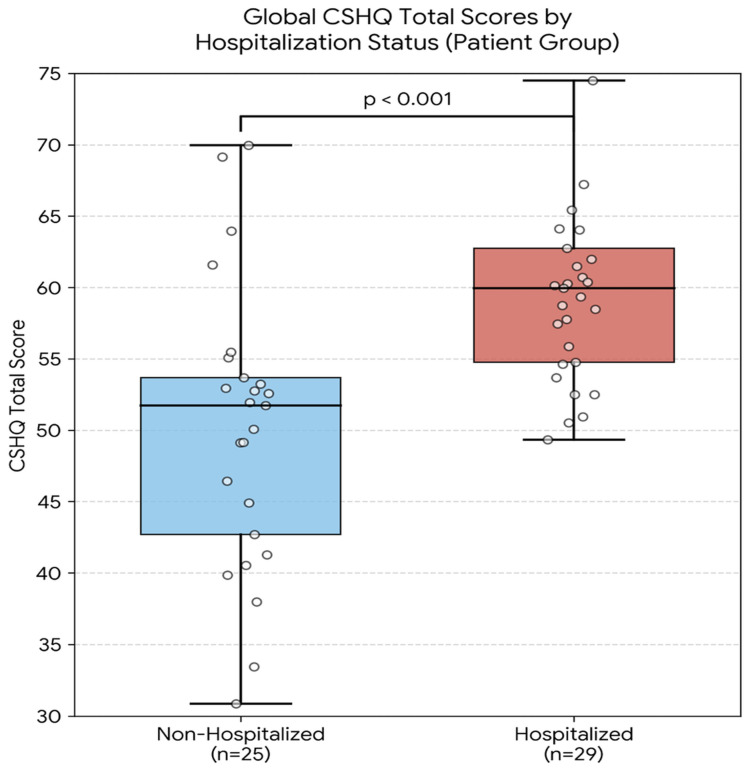
Global CSHQ total scores by hospitalization status within the patient group. Boxplot showing global CSHQ total scores in hospitalized (*n* = 29) and non-hospitalized patients (*n* = 25). Recent hospitalization was associated with significantly higher global sleep disturbance within the patient group (*p* < 0.001).

In a patient-only model including recent hospitalization, age, and sex, recent hospitalization was independently associated with higher global sleep disturbance (B = 11.177, *p* < 0.001), and this model explained 43.3% of the variance (R^2^ = 0.433) (Figure 1).

## 4. Discussion

This study found that children with leukemia and lymphoma had higher parent-reported global sleep disturbance scores than clinically stable children followed for benign hematological conditions. The difference was not distributed uniformly across all sleep domains; rather, it was mainly driven by higher scores in sleep anxiety and parasomnias, whereas sleep duration did not differ significantly between groups. These findings suggest that sleep disturbance in pediatric hematologic malignancies may be better conceptualized as a selective pattern of anxiety-related and nocturnal behavioral sleep difficulties rather than as reduced sleep quantity alone [6,7,8,11,14,15,16].

This distinction is clinically important. In pediatric hematology-oncology care, providers might miss sleep problems in children who seem to get enough sleep in total. However, sleep fragmentation, nighttime fears, and parasomnia-type symptoms might still place a significant strain during daytime on several levels. Our findings therefore support the view that sleep should be considered a meaningful supportive care domain in children with leukemia and lymphoma [1,2,3,4,6,7,8]. An important finding was that recent hospitalization showed the strongest association with global sleep disturbance within the patient group. This association likely reflects more than inpatient location alone; recent hospitalization in this setting may reflect a broader treatment-context burden, including environmental disruption, acute symptom burden, and altered daily routines [10,18,19,20,21,22,23]. Accordingly, our data suggest that sleep disturbance during pediatric leukemia and lymphoma therapy may be closely linked to periods of intensified care rather than attributable to a single isolated exposure.

The absence of a considerable disparity between the off-treatment group and the comparison group may suggest some normalization of sleep disturbance after treatment, but this finding needs to be confirmed by further research. The results from our study, in which recent hospitalization showed the strongest relationship with sleep problems, agree with the broader literature on hospitalization in children, where environmental disruption, nighttime care activities, pain, and treatment-related interruptions are established contributors to poor sleep quality [10,18,19,20,21,22,23]. The threshold-based analysis should be interpreted cautiously. Although a greater proportion of patients exceeded the previously proposed CSHQ screening threshold of ≥ 41, more than three-quarters of the comparison group also exceeded this cutoff. This finding suggests that the threshold is sensitive but not specific for clinically confirmed sleep disorders in this population. Therefore, we did not interpret the cutoff as diagnostic. Instead, the main evidence for between-group differences comes from the continuous CSHQ total score and domain-specific comparisons, which showed a moderate elevation in global sleep burden and selective increases in sleep anxiety and parasomnias among children with leukemia and lymphoma.

Corticosteroid exposure was also associated with higher sleep disturbance in patient-only models that did not include hospitalization. In the present cohort, dexamethasone at 20 mg/m^2^/day was used in leukemia and non-Hodgkin lymphoma patients, whereas prednisolone at 60 mg/m^2^/day was used in Hodgkin lymphoma patients according to the relevant treatment protocols. Although glucocorticoids may plausibly affect sleep through circadian, neuroendocrine, and arousal-related mechanisms [13], several biological pathways may be relevant. Exogenous corticosteroids may bind to glucocorticoid receptors within limbic structures such as the amygdala and hippocampus, potentially dysregulating neurotransmitter signaling and heightening anxiety-like behavior. They may also suppress endogenous melatonin secretion, thereby contributing to sleep fragmentation and parasomnia-type events. These mechanistic pathways suggest that treatment-induced anxiety may act as a direct biological driver of pediatric sleep pathology in this population, in addition to any anxiety attributable to the underlying chronic illness itself. Nonetheless, corticosteroid exposure was highly correlated with intensive therapy and recent hospitalization in this study. For this reason, the association should be interpreted as a treatment-context-related finding rather than evidence of an independent steroid-specific effect. Because hospitalization and corticosteroid exposure could not be modeled jointly, the beta coefficients observed across both patient-only models most plausibly reflect a shared, overlapping “intensive therapy context” rather than isolated, independent biological or environmental effects.

The pattern found specific to this area may help improve clinical assessment. Questions about fear of going to bed, trouble sleeping alone, restlessness during the night, and frequent waking episodes at night might provide more information about the needs of the group than just total sleep time [5,16]. The increased prevalence of sleep anxiety and parasomnias noted in the current patient population is consistent with recent discoveries in pediatric oncology sleep literature. The objective measures such as actigraphy may be less sensitive in assessing behaviorally based sleep problems—such as bedtime resistance or sleep anxiety, where a child may remain physically still—compared to parentally based screening instruments such as CSHQ [16,24]. The difference might explain why some impairments can be identified without any statistically significant differences in total sleep time between groups. Anxiety has been reported to be higher in children suffering from pediatric oncology compared to control subjects; therefore, it is possible to hypothesize a psychological model that explains the occurrence of sleep-related anxiety in the present sample [25].

There was an inverse correlation between male sex and CSHQ in both the full sample and patient subsample regressions. Care should be taken in drawing any conclusions from this result because it was not part of our initial hypotheses and was not intended to explore sex differences among sleep phenotypes. The noted differences could be real in terms of biological sex differences in sleep anxiety or reporting patterns among parents; however, further research using larger samples stratified by sex would be necessary before any firm conclusion is made. Aside from condition-specific variables, parent education was found to be an independent predictor for total sleep disturbance in the entire sample group. For children whose parents had less educated backgrounds, the scores in CSHQ were found to be greater, an outcome consistent with existing literature showing a link between poor education and negative impacts on pediatric sleep outcomes [26]. It is noteworthy that the relationship held even when adjustments were made for patient condition, age, and sex, suggesting that the sociodemographic characteristics at a familial level play a role in sleep burden independent of the medical setting. Several mechanisms may explain this protective association. Parents with higher educational attainment may possess greater health literacy, enabling them to better understand and implement structured pediatric sleep hygiene practices, such as consistent bedtimes and reduced pre-sleep stimulation, even under the strain of a chronic illness. Higher parental education may also serve as a proxy for broader socioeconomic resources, including more stable housing, greater access to caregiving support, and reduced financial strain, all of which could buffer children from environmental stressors that disrupt sleep during cancer treatment. Additionally, more educated parents may communicate more effectively with the medical team regarding sleep-related concerns, facilitating earlier identification and management of sleep problems. These potential pathways warrant further investigation in future studies specifically designed to disentangle health literacy, socioeconomic status, and caregiving practices.

### Strengths and Limitations

The strengths of this study include its focus on an underexplored supportive care domain in pediatric hematologic malignancies, the assessment of both global and domain-specific sleep disturbance, and the inclusion of an age- and sex-matched comparison group. In addition, recent hospitalization and corticosteroid exposure were evaluated separately because these variables were strongly correlated with each other and with the intensive treatment phase.

Several limitations should be acknowledged. First, the comparison group was not a community-based healthy control group. Although these children were clinically stable and followed only for benign hematological conditions, the conditions represented in this group, particularly iron deficiency anemia, are not necessarily neutral with respect to sleep. In addition, unmeasured psychosocial factors associated with outpatient follow-up may have influenced parental reporting. Moreover, because these children were followed for chronic, non-oncological hematological conditions rather than recruited as entirely healthy peers, the comparison group may itself have carried baseline sleep vulnerabilities or psychosocial burden associated with living with a chronic diagnosis, which would be expected to narrow rather than exaggerate the observed between-group difference. In particular, iron deficiency anemia has been directly linked in the pediatric literature to disrupted sleep architecture, increased sleep fragmentation, and dopaminergic motor restlessness resembling restless legs syndrome; such biologically mediated effects could have independently elevated baseline CSHQ scores in the comparison group, making our reported difference a conservative rather than an inflated estimate of the true sleep burden associated with leukemia and lymphoma.

Second, sleep disturbance was assessed using the parent-reported CSHQ rather than objective measures such as actigraphy or polysomnography. Therefore, the findings reflect parent-perceived sleep disturbance burden and cannot distinguish objectively confirmed sleep disruption from parental perceptions of sleep problems. Without such objective diagnostic modalities, this study cannot evaluate actual sleep architecture, macro-structural sleep disruptions, or biological sleep efficiency. Parental psychological distress, anxiety, or treatment-related concerns were not measured and may have influenced CSHQ reporting, particularly in the oncology group. Parents of children undergoing cancer treatment often experience substantial psychological distress, anxiety, and trauma related to the diagnosis and its treatment, and this heightened parental burden could plausibly inflate or otherwise skew subjective reporting of their child’s sleep difficulties independent of the child’s actual sleep state.

Third, the cross-sectional design precludes causal inference. Recent hospitalization, corticosteroid exposure, treatment phase, symptom burden, and treatment intensity were closely interrelated. Therefore, associations involving hospitalization and corticosteroid exposure should be interpreted as treatment-context-related findings rather than independent causal effects.

Fourth, the sample size was fairly small, especially when considering subgroup analysis and regression analysis. As such, multivariate analyses were limited, and subgroup analyses should be considered exploratory in nature. The sample included individuals with different diagnoses such as leukemia, non-Hodgkin’s lymphoma, and Hodgkin’s lymphoma that have different treatment procedures, uses of corticosteroids, and CNS treatments. Household income, room-sharing, and ambient noise level were not collected in this study and represent an additional unmeasured environmental limitation. Pooling these diagnoses, treatment phases, and corticosteroid agents into broad, binary categories was an unavoidable constraint imposed by the exploratory sample size, and this pooling necessarily limits the generalizability of the regression coefficients to specific diagnostic or treatment subgroups. Future studies with a larger sample and objective measurement of sleep and parental stress are needed to corroborate these results. Although there are some shortcomings, this research produces clinically important results, suggesting that sleep problems in children suffering from leukemia and lymphoma are most apparent during periods of intensive treatment and after hospitalization. Such results confirm the assessment of sleep as an element of supportive care when undergoing continuous therapy.

## 5. Conclusions

Leukemia and lymphoma patients experienced more sleep disturbances according to parental reports than those with benign hematological diseases. Sleep disturbance was selective in nature and included sleep anxiety and parasomnias but not decreased sleep time, although these specific domain-level findings are exploratory and require validation in larger, confirmatory cohorts before being considered definitive. Hospitalization turned out to be the most significant clinical factor associated with global sleep burden; nevertheless, one should consider it in relation to the context of intensive therapy, corticosteroid use, and therapy-related interference. Overall, these findings support incorporating sleep-related supportive care assessment into routine clinical follow-up throughout the course of therapy.

## Figures and Tables

**Table 1:** Demographic and clinical characteristics of the study population.

Variable	Patients (*n* = 54)	Comparison Group (*n* = 56)	*p*-Value
Age, years (mean ± SD)	7.58 ± 3.87	8.44 ± 3.52	0.229
Male sex, *n* (%)	27 (50.0%)	27 (48.2%)	1.000
Female sex, *n* (%)	27 (50.0%)	29 (51.8%)	
Parental education, *n* (%)			0.677
Primary school	21 (38.9%)	19 (33.9%)	
Middle school	4 (7.4%)	3 (5.4%)	
High school	11 (20.4%)	17 (30.4%)	
University	18 (33.3%)	17 (30.4%)	
Steroid use, *n* (%)	21 (38.9%)	—	—
Recent hospitalization (preceding 8 weeks), *n* (%)	29 (53.7%)	—	—
Diagnosis, *n* (%)			
Leukemia	32 (59.3%)		
Lymphoma	22 (40.7%)		
Non-Hodgkin lymphoma	16 (29.6%)		
Hodgkin lymphoma	6 (11.1%)		
Treatment phase, *n* (%)			
Intensive therapy	28 (51.9%)	—	—
Maintenance	10 (18.5%)	—	—
Off-treatment	16 (29.6%)	—	—

Values in Table 1 are presented as *n* (%) unless otherwise specified; clinical sub-categorizations (corticosteroid exposure, recent hospitalization, diagnosis, and treatment phase) apply strictly to the patient group.

**Table 2:** Comparison of CSHQ sleep scores between patients and comparison group. Independent samples *t*-test.

Outcome	Patients (*n* = 54) Mean ± SD	Comparison Group (*n* = 56) Mean ± SD	Mean Difference	*p*-Value	Cohen’s d
CSHQ Total Score	53.56 ± 8.28	49.07 ± 8.09	4.49	0.005	0.55
Bedtime Resistance	9.37 ± 2.64	8.98 ± 2.41	0.39	0.39	0.16
Sleep Onset Delay	1.93 ± 0.72	1.75 ± 0.66	0.18	0.16	0.26
Sleep Duration	4.13 ± 1.20	3.95 ± 1.05	0.18	0.34	0.16
Sleep Anxiety	7.81 ± 2.34	6.82 ± 2.11	0.99	0.018	0.45
Night Wakings	3.70 ± 1.45	3.42 ± 1.33	0.28	0.29	0.20
Parasomnias	11.98 ± 3.08	10.41 ± 2.76	1.57	0.006	0.54
Sleep-Disordered Breathing	3.24 ± 1.06	3.08 ± 0.98	0.16	0.39	0.15
Daytime Sleepiness	13.44 ± 3.21	12.91 ± 3.08	0.53	0.35	0.17

CSHQ total and subscale scores are presented as mean ± SD. Higher scores indicate greater sleep disturbance. *p* values were derived from independent samples *t*-tests.

**Table 3:** Multivariable Linear Regression Analyses for Global Sleep Disturbance. Dependent Variable: CSHQ Total Score. Panel A: Whole Sample Model—Extended (*n* = 110) R^2^ = 0.225; Adjusted R^2^ = 0.195; F(4,104) = 7.53; *p* < 0.001. Panel B: Patient-Only Model 1 (Hospitalization Model, *n* = 54). R^2^ = 0.433; Adjusted R^2^ = 0.402; F(3,50) = 12.72; *p* < 0.001. Panel C: Patient-Only Model 2 (Corticosteroid Model, *n* = 54). R^2^ = 0.267; Adjusted R^2^ = 0.223; F(3,50) = 6.07; *p* < 0.001.

**A**					
**Predictor**	**B** **(Unstandardized Coefficient)**	**SE**	**95% CI**	**T**	***p***
Intercept	58.072	2.667	52.783–63.361	21.77	<0.001
Age	**−0.238**	0.201	−0.636–0.160	−1.19	0.239
Male sex (M vs. F)	−3.166	1.472	−6.084–−0.247	−2.15	0.034
Patient status (Patient vs. Control)	**4.158**	1.471	1.242–7.075	2.83	0.006
Parental education	−2.131	0.583	−3.287–−0.976	−3.66	<0.001
**B**					
Intercept	47.517	2.314	42.869–52.165	20.54	<0.001
Hospitalization (Yes vs. No)	11.177	1.859	7.443–14.911	6.01	<0.001
Age	0.319	0.229	−0.141–0.779	1.39	0.170
Male sex	−4.766	1.859	−8.500–−1.032	−2.56	0.013
**C**					
Intercept	50.477	2.522	45.411–55.543	20.02	<0.001
Steroid (Yes vs. No)	9.543	2.344	4.835–14.251	4.07	<0.001
Age	0.289	0.260	−0.233–0.811	1.11	0.272
Male sex (M vs. F)	−5.656	2.292	−10.260–−1.052	−2.47	0.017

Panel A shows the whole-sample model, whereas Panels B and C are restricted to the patient group. Regression coefficients are reported as unstandardized B values. Hospitalization and corticosteroid exposure were modeled separately because of strong collinearity.

## Data Availability

The datasets generated during and/or analyzed during the current study are available from the corresponding author on reasonable request.

## Abbreviations

ANOVA	Analysis of variance
CI	Confidence interval
CSHQ	Children’s Sleep Habits Questionnaire
OR	Odds ratio
SD	Standard deviation
SE	Standard error
SPSS	Statistical Package for the Social Sciences
